# Supplementary material for: Resolving the full spectrum of human genome variation using Linked-Reads
Source: Genome Res. 2019 Apr;29(4):635–45. doi: 10.1101/gr.234443.118 (PMC6442396; doi:10.1101/gr.234443.118)
Supplement: Supplemental Material [file supp_gr.234443.118_Supplemental_longranger-2.2.zip › longranger-2.2.tar.bz2!/lr2.2-public/lib/stan/cmdstan/stan/doc/api/html/agrad_2rev_2functions_2ibeta_8hpp_source.html]

Stan: src/stan/agrad/rev/functions/ibeta.hpp Source File


|  |  |
| --- | --- |
|  | Stan  2.5.0  probability, sampling & optimization |


- Main Page
- Related Pages
- Namespaces
- Classes
- Files

- File List
- File Members

All Classes Namespaces Files Functions Variables Typedefs Enumerations Enumerator Friends Macros Pages

- src
- stan
- agrad
- rev
- functions

ibeta.hpp

Go to the documentation of this file.

1 #ifndef STAN\_\_AGRAD\_\_REV\_\_FUNCTIONS\_\_IBETA\_HPP

2 #define STAN\_\_AGRAD\_\_REV\_\_FUNCTIONS\_\_IBETA\_HPP

3

4 #include <boost/math/special\_functions/digamma.hpp>

5 #include <boost/math/special\_functions/gamma.hpp>

6 #include <stan/agrad/rev/var.hpp>

7 #include <stan/agrad/rev/internal/vvv\_vari.hpp>

8 #include <stan/agrad/rev/internal/vvd\_vari.hpp>

9 #include <stan/agrad/rev/internal/vdv\_vari.hpp>

10 #include <stan/agrad/rev/internal/vdd\_vari.hpp>

11 #include <stan/agrad/rev/internal/dvv\_vari.hpp>

12 #include <stan/agrad/rev/internal/dvd\_vari.hpp>

13 #include <stan/agrad/rev/internal/ddv\_vari.hpp>

14 #include <stan/math/functions/ibeta.hpp>

15

16 namespace stan {

17  namespace agrad {

18

19  namespace {

25  double ibeta\_hypergeometric\_helper(double a, double b, double z, double precision=1e-8, double max\_steps=1000) {

26  double val=0;

27  double diff=1;

28  double k=0;

29  double a\_2 = a\*a;

30  double bprod = 1;

31  while (std::abs(diff) > precision && (++k < max\_steps) && !std::isnan(diff)) {

32  val += diff;

33  bprod \*= b+k-1.0;

34  diff = a\_2\*std::pow(a+k,-2)\*bprod\*std::pow(z,k)/boost::math::tgamma(k+1);

35  }

36  return val;

37  }

38

39  class ibeta\_vvv\_vari : public op\_vvv\_vari {

40  public:

41  ibeta\_vvv\_vari(vari\* avi, vari\* bvi, vari\* xvi) :

42  op\_vvv\_vari(stan::math::ibeta(avi->val\_,bvi->val\_,xvi->val\_),avi,bvi,xvi) {

43  }

44  void chain() {

45  double a = avi\_->val\_;

46  double b = bvi\_->val\_;

47  double c = cvi\_->val\_;

48

49  using std::sin;

50  using std::pow;

51  using std::log;

52  using boost::math::constants::pi;

53  using boost::math::tgamma;

54  using boost::math::digamma;

55  using boost::math::ibeta;

56  using stan::agrad::ibeta\_hypergeometric\_helper;

57  avi\_->adj\_ += adj\_ \*

58  (log(c) - digamma(a) + digamma(a+b)) \* val\_ -

59  tgamma(a)\*tgamma(a+b)/tgamma(b) \* pow(c,a) / tgamma(1+a) / tgamma(1+a) \* ibeta\_hypergeometric\_helper(a, 1-b, c);

60  bvi\_->adj\_ += adj\_ \*

61  (tgamma(b)\*tgamma(a+b)/tgamma(a)\*pow(1-c,b) \* ibeta\_hypergeometric\_helper(b,1-a,1-c)/tgamma(b+1)/tgamma(b+1)

62  + ibeta(b, a, 1-c) \* (digamma(b) - digamma(a+b) - log(1-c)));

63  cvi\_->adj\_ += adj\_ \*

64  boost::math::ibeta\_derivative(a,b,c);

65  }

66  };

67  class ibeta\_vvd\_vari : public op\_vvd\_vari {

68  public:

69  ibeta\_vvd\_vari(vari\* avi, vari\* bvi, double x) :

70  op\_vvd\_vari(stan::math::ibeta(avi->val\_,bvi->val\_,x),avi,bvi,x) {

71  }

72  void chain() {

73  double a = avi\_->val\_;

74  double b = bvi\_->val\_;

75  double c = cd\_;

76

77  using std::sin;

78  using std::pow;

79  using std::log;

80  using boost::math::constants::pi;

81  using boost::math::tgamma;

82  using boost::math::digamma;

83  using boost::math::ibeta;

84  using stan::agrad::ibeta\_hypergeometric\_helper;

85  avi\_->adj\_ += adj\_ \*

86  (log(c) - digamma(a) + digamma(a+b)) \* val\_ -

87  tgamma(a)\*tgamma(a+b)/tgamma(b) \* pow(c,a) / tgamma(1+a) / tgamma(1+a) \* ibeta\_hypergeometric\_helper(a, 1-b, c);

88  bvi\_->adj\_ += adj\_ \*

89  (tgamma(b)\*tgamma(a+b)/tgamma(a)\*pow(1-c,b) \* ibeta\_hypergeometric\_helper(b,1-a,1-c)/tgamma(b+1)/tgamma(b+1)

90  + ibeta(b, a, 1-c) \* (digamma(b) - digamma(a+b) - log(1-c)));

91  }

92  };

93  class ibeta\_vdv\_vari : public op\_vdv\_vari {

94  public:

95  ibeta\_vdv\_vari(vari\* avi, double b, vari\* xvi) :

96  op\_vdv\_vari(stan::math::ibeta(avi->val\_,b,xvi->val\_),avi,b,xvi) {

97  }

98  void chain() {

99  double a = avi\_->val\_;

100  double b = bd\_;

101  double c = cvi\_->val\_;

102

103  using std::sin;

104  using std::pow;

105  using std::log;

106  using boost::math::constants::pi;

107  using boost::math::tgamma;

108  using boost::math::digamma;

109  using boost::math::ibeta;

110  using stan::agrad::ibeta\_hypergeometric\_helper;

111  avi\_->adj\_ += adj\_ \*

112  (log(c) - digamma(a) + digamma(a+b)) \* val\_ -

113  tgamma(a)\*tgamma(a+b)/tgamma(b) \* pow(c,a) / tgamma(1+a) / tgamma(1+a) \* ibeta\_hypergeometric\_helper(a, 1-b, c);

114  cvi\_->adj\_ += adj\_ \*

115  boost::math::ibeta\_derivative(a,b,c);

116  }

117  };

118  class ibeta\_vdd\_vari : public op\_vdd\_vari {

119  public:

120  ibeta\_vdd\_vari(vari\* avi, double b, double x) :

121  op\_vdd\_vari(stan::math::ibeta(avi->val\_,b,x),avi,b,x) {

122  }

123  void chain() {

124  double a = avi\_->val\_;

125  double b = bd\_;

126  double c = cd\_;

127

128  using std::sin;

129  using std::pow;

130  using std::log;

131  using boost::math::constants::pi;

132  using boost::math::tgamma;

133  using boost::math::digamma;

134  using boost::math::ibeta;

135  using stan::agrad::ibeta\_hypergeometric\_helper;

136  avi\_->adj\_ += adj\_ \*

137  (log(c) - digamma(a) + digamma(a+b)) \* val\_ -

138  tgamma(a)\*tgamma(a+b)/tgamma(b) \* pow(c,a) / tgamma(1+a) / tgamma(1+a) \* ibeta\_hypergeometric\_helper(a, 1-b, c);

139  }

140  };

141  class ibeta\_dvv\_vari : public op\_dvv\_vari {

142  public:

143  ibeta\_dvv\_vari(double a, vari\* bvi, vari\* xvi) :

144  op\_dvv\_vari(stan::math::ibeta(a,bvi->val\_,xvi->val\_),a,bvi,xvi) {

145  }

146  void chain() {

147  double a = ad\_;

148  double b = bvi\_->val\_;

149  double c = cvi\_->val\_;

150

151  using std::sin;

152  using std::pow;

153  using std::log;

154  using boost::math::constants::pi;

155  using boost::math::tgamma;

156  using boost::math::digamma;

157  using boost::math::ibeta;

158  using stan::agrad::ibeta\_hypergeometric\_helper;

159  bvi\_->adj\_ += adj\_ \*

160  (tgamma(b)\*tgamma(a+b)/tgamma(a)\*pow(1-c,b) \* ibeta\_hypergeometric\_helper(b,1-a,1-c)/tgamma(b+1)/tgamma(b+1)

161  + ibeta(b, a, 1-c) \* (digamma(b) - digamma(a+b) - log(1-c)));

162  cvi\_->adj\_ += adj\_ \*

163  boost::math::ibeta\_derivative(a,b,c);

164  }

165  };

166  class ibeta\_dvd\_vari : public op\_dvd\_vari {

167  public:

168  ibeta\_dvd\_vari(double a, vari\* bvi, double x) :

169  op\_dvd\_vari(stan::math::ibeta(a,bvi->val\_,x),a,bvi,x) {

170  }

171  void chain() {

172  double a = ad\_;

173  double b = bvi\_->val\_;

174  double c = cd\_;

175

176  using std::sin;

177  using std::pow;

178  using std::log;

179  using boost::math::constants::pi;

180  using boost::math::tgamma;

181  using boost::math::digamma;

182  using boost::math::ibeta;

183  using stan::agrad::ibeta\_hypergeometric\_helper;

184  bvi\_->adj\_ += adj\_ \*

185  (tgamma(b)\*tgamma(a+b)/tgamma(a)\*pow(1-c,b) \* ibeta\_hypergeometric\_helper(b,1-a,1-c)/tgamma(b+1)/tgamma(b+1)

186  + ibeta(b, a, 1-c) \* (digamma(b) - digamma(a+b) - log(1-c)));

187  }

188  };

189  class ibeta\_ddv\_vari : public op\_ddv\_vari {

190  public:

191  ibeta\_ddv\_vari(double a, double b, vari\* xvi) :

192  op\_ddv\_vari(stan::math::ibeta(a,b,xvi->val\_),a,b,xvi) {

193  }

194  void chain() {

195  double a = ad\_;

196  double b = bd\_;

197  double c = cvi\_->val\_;

198

199  cvi\_->adj\_ += adj\_ \*

200  boost::math::ibeta\_derivative(a,b,c);

201  }

202  };

203  }

204

223  inline var ibeta(const var& a,

224  const var& b,

225  const var& x) {

226  return var(new ibeta\_vvv\_vari(a.vi\_, b.vi\_, x.vi\_));

227  }

228

229  }

230 }

231 #endif

vvd\_vari.hpp

stan::agrad::sin

fvar< T > sin(const fvar< T > &x)

**Definition:** sin.hpp:14

stan::math::ibeta

double ibeta(const double a, const double b, const double x)

The normalized incomplete beta function of a, b, and x.

**Definition:** ibeta.hpp:23

dvd\_vari.hpp

stan::agrad::tgamma

fvar< T > tgamma(const fvar< T > &x)

**Definition:** tgamma.hpp:15

stan::agrad::pow

fvar< T > pow(const fvar< T > &x1, const fvar< T > &x2)

**Definition:** pow.hpp:17

vdd\_vari.hpp

ibeta.hpp

stan::agrad::var::vi\_

vari \* vi\_

Pointer to the implementation of this variable.

**Definition:** var.hpp:40

vvv\_vari.hpp

stan::agrad::ibeta

var ibeta(const var &a, const var &b, const var &x)

The normalized incomplete beta function of a, b, and x.

**Definition:** ibeta.hpp:223

stan::agrad::abs

fvar< T > abs(const fvar< T > &x)

**Definition:** abs.hpp:19

var.hpp

dvv\_vari.hpp

stan::agrad::var

Independent (input) and dependent (output) variables for gradients.

**Definition:** var.hpp:27

stan::math::e

double e()

Return the base of the natural logarithm.

**Definition:** constants.hpp:86

stan::agrad::digamma

fvar< T > digamma(const fvar< T > &x)

**Definition:** digamma.hpp:16

vdv\_vari.hpp

std::isnan

int isnan(const stan::agrad::var &a)

Checks if the given number is NaN.

**Definition:** numeric\_limits.hpp:62

stan::math::pi

double pi()

Return the value of pi.

**Definition:** constants.hpp:77

ddv\_vari.hpp

stan::agrad::log

fvar< T > log(const fvar< T > &x)

**Definition:** log.hpp:15


---

[ Stan Home Page ]

*© 2011–2014,
Stan Development Team.*
